# BdorCSP2 Is Important for Antifeed and Oviposition-Deterring Activities Induced by Rhodojaponin-III against *Bactrocera dorsalis*

**DOI:** 10.1371/journal.pone.0077295

**Published:** 2013-10-14

**Authors:** Xin Yi, Haiming Zhao, Xiaolin Dong, Peidan Wang, Meiying Hu, Guohua Zhong

**Affiliations:** Laboratory of Insect Toxicology, Key Laboratory of Pesticide and Chemical Biology, Ministry of Education, South China Agricultural University, Guangzhou, People’s Republic of China; University of Copenhagen, Denmark

## Abstract

Rhodojaponin-III is a nonvolatile botanical grayanoid diterpene compound, which has antifeedant and oviposition deterrence effects against many kinds of insects. However, the molecular mechanism of the chemoreception process remains unknown. In this study, the important role of BdorCSP2 in the recognition of Rhodojaponin-III was identified. The full length cDNA encoding BdorCSP2 was cloned from legs of *Bactrocera dorsalis*. The results of expression pattern revealed that BdorCSP2 was abundantly expressed in the legs of adult *B. dorsalis*. Moreover, the expression of BdorCSP2 could be up-regulated by Rhodojaponin-III. In order to gain comprehensive understanding of the recognition process, the binding affinity between BdorCSP2 and Rhodojaponin-III was measured by fluorescence binding assay. Silencing the expression of BdorCSP2 through the ingestion of dsRNA could weaken the effect of oviposition deterrence and antifeedant of Rhodojaponin-III. These results suggested that BdorCSP2 of *B. dorsalis* could be involved in chemoreception of Rhodojaponin-III and played a critical role in antifeedant and oviposition behaviors induced by Rhodojaponin-III.

## Introduction

Insects use chemical signals to locate and access habitat, food, partners, and sites for oviposition [1]. The selections of feeding and oviposition sites on host plants by insects are important for the survival of their offspring [2]. Previous studies reported that the semiochemicals, which were emitted by plants, played significant roles in the interaction between herbivores and host plants [3]. The compounds which have effects of antifeedant and oviposition deterrence are parts of these defensive chemicals. Researchers are devoted to extract the novel botanical compounds that dually had both antifeeding and oviposition deterring activities to mediate insect behaviors [4–6]. Rhodojaponin-III, a diterpene compound isolated from the flowers of *Rhododendron molle* G, has been reported to have intense antifeedant activities against many kinds of agricultural pests [7]. Besides, unlike other volatile chemicals, Rhodojaponin-III is a nonvolatile compound that could inhibit oviposition activities [7–9]. Consequently, versatile actions of Rhodojaponin-III enabled it to be a promising commercial insecticide. The oriental fruit fly, *Bactrocera dorsalis* (Hendel), is one of the most destructive pests by causing serious financial losses to more than 150 crop species [10]. Thus, there is an urgent need to develop new pest control strategies against *B. dorsalis*. With its intense oviposition deterrence and antifeedant activities, Rhodojaponin-III could be an efficient candidate for sustainable pest management [11]. However, little is known about the detailed chemoreception mechanism of Rhodojaponin-III as an antifeedant and oviposition deterrent to influence insect behaviors. 

The insects could detect external stimuli through hair-like sensilla on the chemosensory organs, such as antenna, maxillary and tarsus [12]. More specifically, insects use olfaction system to search and orientate for locations of potential host plants [13], and then after landing, contacting chemoreception could provide the primary basis for host plant selection and discrimination [14]. Researches showed that flies made contact with the substrate by their tarsi at first, and then reflexively extended their mouthparts if they landed on anything edible [15]. Contact chemosensillas presented on tarsi have been shown to be involved in perceiving non-volatile chemicals and the selections of oviposition sites [16,17]. Therefore, tarsus is crucial in food searching and oviposition sites. However, in contrast to our well-known physiological knowledge of sensilla on antenna, little is known about the functions of chemoreceptors on tarsi. 

Recent studies indicated that chemosensory proteins (CSPs) were very abundant in the outer lymph of contact sensilla in the chemoreception organs, which suggested CSPs could be involved in contact chemoreception [18]. The first CSP was found in *Drosophila melanogaster* and called OS-D (olfactory specific-protein type D) or A10 [19,20]. Since then, many CSPs have been identified in many insect species [18,21,22], and different CSPs were localized in different organs. For example，in locusts, CSPs were found in different tissues, including antennae, thorax, abdomen, head and legs [23]. In *Bombyx mori*, a DIG-RNA probe encoding the BmorCSP1 hybridized not only with mRNAs from male and female antennae but also with mRNAs from legs and other parts of the insect body. Northern blot analysis revealed that high level of BmorCSP2 expressed in legs [24–26]. The CSP of *Cactoblastis cactorum* was isolated from labial palps [27]. The immunolocalization experiment showed the presence of CSPs in the cuticle of pulvilli on the ventral surface of tarsi [28]. Due to the localizations, these proteins have been considered to be involved in chemo-perception and chemical signal transduction [18,29]. However, the exact physiological functions of these proteins have not been fully addressed. In recent years, their abilities to bind small chemicals have been used as tools to understand their physiological functions. Studies confirmed that CSPs were capable of binding a range of aliphatic compounds, esters and other long chain compounds [30–32]. The *Adelphocoris lineolatus* CSP showed high binding affinity with several host-related semiochemicals [17]. CSP of the carpenter ant, *Camponotus japonicus*, could bind with cuticular hydrocarbons, which could function as contacting pheromones for nest-mate discrimination [33]. Additionally, CSP was demonstrated to be able to bind Rhodojaponin-III with good affinity in *Plutella xylostella* [34] and *Spodoptera litura* [35]. These fundamental discoveries not only provided direct evidence that CSPs could be extended to be carrier proteins for various ligands, but also indicated that they may play roles in recognizing nonvolatile compounds and carrying contact chemical stimuli to the receptors.

In this paper, the existence of a specific CSP gene from the legs of *B. dosrsalis* was reported and its expression profile was characterized. In order to provide the direct evidence to elucidate the chemoreception process of Rhodojaponin-III in insect, the interaction between BdorCSP2 and Rhodojaponin-III was identified by the binding assay *in vitro* and the behavioral analysis *in vivo*. To examine the involvement of BdorCSP2 in antifeedant and oviposition deterrence effects of Rhodojaponin-III, RNA interference (RNAi) technique was applied to silence the expression of BdorCSP2 by feeding the insects with artificial diet containing dsRNA. Collectively, our data suggested that CSP-dependent chemical recognition was critical for *B. dorsalis* to identify the nonvolatile insecticide Rhodojaponin-III.

## Materials and Methods

### Ethics statement

The animal study proposal was approved by the Institutional Animal Care and Use Committee (IACUC) of the South China Agricultural University with the permit number: SCAU/IACUC2012-067. All the rabbit experimental procedures were performed in accordance with the Regulations for the Administration of Affairs Concerning Experimental Animals approved by the State Council of People’s Republic of China. We confirmed that the studies did not involve endangered or protected species.

### Insects

The oriental fruit flies, *B. dorsalis* (Hendel), were maintained in our laboratory at 28 °C, using 70% RH and a 14: 10 h light: dark photoperiod. Adult flies were reared on artificial diets consisting of yeast extract, sugar, honey, and agar [36,37].

### Antenna wax sealed in the repellent assay

Rhodojaponin-III (min.95% AI) was extracted and purified by the method of Klocke [38] and stored in the laboratory of insect toxicology (South China Agricultural University). The standard was obtained kindly from the Utah Natural Products Research Institute. Rhodojaponin-III was diluted to final concentration of 100 mg/L. The bananas were covered with Rhodojaponin-III evenly, while equal volume of water was used as control. After blow-drying the solvent, one banana was placed into a 30 cm× 30 cm× 30 cm wooden box. To insulate antennas, the thin coating of paraffin oil was applied to antennas with a soft brush before the flies were placed in the box. Then, 20 antennas sealed mature flies (female : male = 1 : 1) were placed in the box with one Rhodojaponin-III treated banana inside (untreated banana was used as control). The oviposition deterrence rate of the unsealed flies was tested under the same condition. Each experiment was performed in triplicate. The number of eggs in bananas was recorded after 48 h treatment. The oviposition rate was calculated by the fomula:

Oviposition deterrence rate (%)= (A-B)/(A+B)× 100

(A: The number of eggs on the untreated banana, B: The number of eggs on the Rhodojaponin-III treated banana.)

### Sample preparation and RNA isolation

For the expression profile of developmental stages, the samples were extracted from egg, first instar larva, second instar larva, third instar larva, 1 d-old pupa, 4 d-old pupa, 7 d-old pupa, 10 d-old pupa, newly-emerged male adult and newly-emerged female adults. Additionally, samples from different organs and tissues were isolated, including antennas, heads (with antennae removed), thoraxs, fore legs, middle legs, hind legs, wings and genital segments. The samples were excised at the base and immediately transferred into eppendorf tubes immersed in liquid nitrogen. All tissues were stored at -80 °C until being used experimentally.

Total RNA from different samples was extracted using the E.Z.N.A.^TM^ total RNA isolation system kit (Omega, USA) according to the manufacturer's instructions. The isolated RNA was reversely transcribed to first-strand cDNA with M-MLV reverse transcriptase (TaKaRa, China) and oligo(dT)_18_ as primer at 42 °C for 60 min. The reaction was terminated by heating at 95 °C for 5 min, and the products were stored at -20 °C.

### Cloning of BdorCSP2 cDNA and sequence analysis

The samples were extracted from legs of *B. dorsalis*. Based on the conserved sequences of CSPs from contacting chemosensory organs, the partial sequence of BdorCSP2 was amplified by the degenerate primers CSPF and CSPR (Table1). The GeneRacer Kit (Clontech, US) was used to gain the full sequence of BdorCSP2. The primers CSP-3RACEouter, CSP-3RACEInner and CSP-5RACEouter, CSP-5RACEInner (Table 1) were used for the amplification of the 3' RACE (rapid-amplification of cDNA ends) and 5' RACE. The primers CSP-3RACEouter, CSP-5RACEouter and Universal primer Mix (UPM, Clontech) were used to carry out first round PCR. With the first round PCR product as template, nest PCR was carried out with the primers CSP-3RACEinner, CSP-5RACEinner and the Nested Universal Primer (NUP, Clontech). Amplifications were carried out according to the manufacturer's protocol. Sequences were cloned into pMD20-T vector (TaKaRa, China) and sequenced completely in both directions. The sequence identified from contact sensilla was aligned using Clustal W software package [39]. The neighbor-joining tree was constructed using MEGA 4.0 (Molecular Evolutionary Genetics Analysis, Version 4.0) and assessed by the bootstrap test based on 1, 000 replicates.

**Table 1:** Primers used in this study.

primers	Primer sequence
*Dgenerate primers*	
CSPF	5′-TAYGAYAAYGTBAAYGTGGAYG-3′
CSPR	5′-AARAARATHYTNCCCGACGC-3′
*For RACE*	
CSP-3RACEOuter	5′-CAACAGTGGAGAATCTCACAGC-3′
CSP-3RACEInner	5′- GTATGGATGAAACTAGGGAACA-3′
CSP-5RACEOuter	5′-TTATGGTTCAGGCTTGCGTCTTAG-3′
CSP-5RACEInner	5′-GCTTGCGTCTTAGTAACCCTTC-3′
*For RT-PCR and RT-qPCR*	
Bdor-CSP-F	5′- ATGTTGCGCTTCGTAGCCGCTTCGG-3′
Bdor-CSP-R	5’-TTAGTAACCGCTTCCGTAGTTTTTCTC-3’
Actin-F	5′- ATCTGGCATCACACTTTCTAC -3′
Actin-R	5′- GTCATCTTCTCACGGTTAGC-3′
*For Bdor-CSP dsRNA synthesis*	
T7Bdor-CSPF	5′- GGATCCTAATACGACTCACTATAGGTGCTGATTTGCGTCGTTT -3′
Bdor-CSPR	5′- GCCATCGCCTTTGTCCTT -3′
Bdor-CSPF	5’- TGCTGATTTGCGTCGTTT-3’
T7 Bdor-CSPR	5‘-GGATCCTAATACGACTCACTATAGGGCCATCGCCTTTGTCCTT-3’
*For GFP dsRNA synthesis*	
T7GFP-F	5’-GGATCCTAATACGACTCACTATAGGT AAGGGCGAGGAGCTGTTCACCG-3’
GFP-R	5‘-CAGCAGGACCATGTGATCGCG-3’
T7GFP-F	5′-AAGGGCGAGGAGCTGTTCACCG-3′
GFP-R	5′-GGATCCTAATACGACTCACTATAGGTCAGCAGGACCATGTGATCGCG-3′

### Expression patterns of BdorCSP2

The expression patterns of BdorCSP2 were further investigated by real-time PCR (RT-PCR) and quantitative real-time PCR (qRT-PCR). qRT-PCR was performed using iCycler iQ Real-Time PCR Detection System (Bio-Rad) with SYBR green dye (Taraka, China) binding to double-strand DNA at the end of each elongation cycle. Amplification process was carried out using the primers: Bdor-CSP-F and Bdor-CSP-R (Table 1). For internal standardization, actin was amplified with the primers: Actin-F and Actin-R (Table 1). To make sure the reproducibility, amplifications were performed in triplicates. The relative gene expression datum were analyzed using the 2^-△△CT^ method as described by Livak [40]. 

For RT-PCR analysis, amplification was performed using the same primes as qRT-PCR by denaturing at 94 °C for 5 min, followed by 27 cycles of 94 °C for 30 s, 60 °C for 30 s and 72 °C for 45 s, with a final extension at 72 °C for 10 min. PCR products were analyzed on 1.2% agarose gels.

### Rhodojaponin-III regulation

A total of 80 mature flies (female : male = 1 : 1) were starved for 12 h and then placed in a 30 cm× 30 cm× 30 cm wooden box. The bananas were covered with Rhodojaponin-III evenly, while equal volume of water was used as control. After blow-drying the solvent, one banana was placed into one box. Total RNA was isolated from the legs after being treated for 12 h and 24 h, respectively, and reversely transcribed into first-strand cDNAs using M-MLV Reverse Transcriptase. The expression level of BdorCSP2 after Rhodojaponin-III treatment was analyzed by qRT-PCR. 

### Expression of recombinant and polyclonal antibody production for BdorCSP2

The sequence encoding of mature BdorCSP2 with EcoR I and Xho I was cloned into pET28a (Invitrogen, US) with T4 DNA ligase (Takara, China) at 14 °C, and then transformed to BL21 (DE3) competent cells (Takara, China). After the positive clone was identified, the single bacterial colony was then inoculated in liquid LB at 37 °C until its OD_600_ reached 0.4-0.6. Isopropyl-D-thiogalactoside (IPTG) was added and then incubated for another 12 h at 28 °C. The cultures were harvested by centrifugation and lysed by the lysis solution (10 mM imidazole, 300 mM NaCl and 50 mM NaH_2_PO_4_). The protein was purified by Ni-NTA His-Bind Resin (Novagen, US). Purified recombinant BdorCSP2 protein was used to immunize rabbit as described previously [41]. The sera of the immunized rabbit was collected as anti-BdorCSP2 sera. The serum titer was shown to have an enzyme linked immunosorbent assay (ELISA) end point of 1:12, 000 using the method of indirect ELISA [42].

### Spectral experimental procedure

In order to measure the conformation of BdorCSP2, its secondary structure was determined by circular dichroism (CD). The CD spectrum was measured on a JASCO J-715 CD spectropolarimeter with 0.25 μM of protein in 50 mM Na phosphate, pH 7.4, at room temperature between 190 and 260 nm. Purified protein with correct secondary structure was used in the forthcoming fluorescence binding assays.

Fluorescence quenching method was used to evaluate the interaction between Rhodojaponin-III and BdorCSP2 according to method of Lartigue and Zhang [32,43]. The fluorescence spectra were recorded on an F-4500 FL Fluorescence Spectrophotometer (HITACHI) at 23 °C. To estimate the binding affinity of Rhodojaponin-III to BdorCSP2 in competitive binding experiment, N-phenyl-1- naphthylamine (1-NPN) was used as the fluorescent probe. Rhodojaponin-III and 1-NPN, which were used in competition experiments were dissolved in HPLC purity grade methanol. The quenching of intrinsic fluorescence was measured with 2 μM BdorCSP2 protein in 50 mM Tris-HCl buffer (pH 7.4) in the presence of 1-NPN at concentrations of 0, 2, 4, 8, 12, 16 and 20 μM, respectively. The excitation wavelength was 295 nm and the emission spectrum was recorded between 300 and 550 nm (slit width of 5 nm was used for both excitation and emission). To measure the affinity of 1-NPN to Bdor-CSP2, the fluorescence of 2 μM 1-NPN in 50 mM Tris-HCl was excited at 337 nm and emission spectra was recorded between 350 nm and 550 nm. And then, 2 μM of protein was added and titrated with aliquots of 1 mM 1-NPN to final concentrations of 2 to 16 μM. The affinity of Rhodojaponin-III and BdorCSP2 was measured by competitive binding assays in presence of both Bdor-CSP2 protein and 1-NPN at 2 μM by adding Rhodojaponin-III from 0 to 200 μM. All values reported were obtained from three independent measurements.

The corresponding maximum fluorescence emission was plotted against the Rhodojaponin-III concentrations for the determination of the binding constant. The amount of bound Rhodojaponin-III was measured using the values of fluorescence intensity with a stoichiometry of 1:1 protein : ligand. The curve was linearized by Scatchard plots. The dissociation constant of the competitor was calculated using the corresponding IC_50_ values (the concentration of the ligand that yielded 50% of the initial fluorescence value) according to the equation: K_d_ = [IC_50_]/(1 + [1-NPN]/K_1-NPN_), where [1-NPN] is the free concentration of 1-NPN and K_1-NPN_ is the dissociation constant of the complex protein/1-NPN.

### RNA interference and bioassay

According to the manufacturer recommendations of T7 Ribo-MAX™ Express RNAi System (Promega, US), two pairs of primers (T7Bdor-CSPF and Bdor-CSPR, Bdor-CSPF and T7 Bdor-CSPR) (Table 1) were designed to synthesize the 366-bp (72-433 bp) region of the BdorCSP2 gene that included a T7 promoter region in the both sense and antisense strands to attach the T7 promoter recognition sites. The GFP gene was used as a control. After the PCR products were obtained, purified DNA from GFP and BdorCSP2 were derived using the T7 Ribomax™ Express RNAi System (Promega, US). The final dsRNA products corresponding to the BdorCSP2 gene (dsBdorCSP2) and GFP gene (dsGFP) were eluted into DEPC water, stored at -80 °C and used within 1 week.

To demonstrate the role of BdorCSP2 in Rhodojaponin-III perception, newly emerged flies (within 12 h after eclosion) were collected and placed into boxes [44]. The feeding procedure for the flies was performed as reported in previous studies [36,44]. The dsRNA solution was added into the artificial diet which was cut into round pieces with a diameter of 3.2 cm. Each piece was covered with 400 μl dsRNA of BdorCSP2 (dsBdorCSP, 1μg/μl). Control groups received equal volume of dsRNA of GFP (dsGFP, 1μg/μl) and DEPC water. The artificial diet with dsRNA and control were renewed every two days. The rearing experiments were carried out as described previously. Each group had 60 individuals with three replicates. To investigate the efficiency of interference in *B. dorsalis*, 10 flies were selected randomly at 2, 4 and 6 d after the ingestion and detected by qRT-PCR. Additionally, the BdorCSP2 protein collected 6 d after the ingestion was investigated by Western blot.

For antifeedant test, after the flies were fed by the dsRNA diet for continuous 14 d, one mature fly was placed in one box with one Rhodojaponin-III treated banana inside (water was used as control), and the experiment was repeated 9 times. The time of feeding activities was recorded in 24 h (night observation was conducted under a dim light). The obtained datum were processed into four factors, including times of feeding activities, the average time of feedings, times of interval within feeding and sustained time of the interval. For oviposition test, 20 mature flies (male: female=1:1), which were fed by the dsRNA diet, were reared in one box with one Rhodojaponin-III treated banana inside (water was used as control), and the experiment was performed in triplicate. The number of eggs on bananas was recorded after 48 h treatment and the oviposition rate was calculated by the formula described above.

### Western blot

Western-blotting analysis was modified according to the methods previously described [45]. Samples were electrophoresed on 12% SDS polyacrylamide mini-gels and transferred to PVDF membranes using Tris–glycine transfer buffer on a mini-Trans-Blot electrophoretic transfer tank (Bio-RAD, USA). Blots were blocked in TBS (100 mM Tris–HCl, pH 7.5, 0.9% NaCl) containing 5% nonfat powdered milk and 0.1% Tween-20 for 1 h. The immunoreactivity was tested with the anti-BdorCSP2 serum (diluted 1: 5000). Blots were washed with TBST three times, each for 5 min. An IgG anti-rabbit antibody, which was conjugated with HRP, was used as a secondary antibody (Tiangen, China) and finally visualized by ECL (enhanced chemiluminescence).

### Data analysis

Data are expressed as the means ± S.E.M of three independent experiments. All the results from experimental replicates were analyzed by one-way analysis of variance (ANOVA) and *t*-test using SPSS 17.0 software (IBM Corporation, Somers, NY).

## Results

### The effect of sealed antennae on oviposition deterring activities of Rhodojaponin-III against *B. dorsalis*

After treatment for 48 h, in the unsealed group, the number of eggs on 100 mg/L Rhodojaponin-III treated banana was significantly less than the control group. The results in Table 2 showed that Rhodojaponin-III had intense oviposition deterring activities against flies. To investigate the role of antennas in the oviposition deterrence activities of Rhodojaponin-III against *B.dorsali*, the antennas were insulated with paraffin oil. Compared to unsealed flies, the flies with antennas sealed showed no significant change in oviposition inhibition rate (Table 2). These results showed that the Rhodojaponin-III still has significant oviposition deterring activities against *B.dorsalis* with sealed antennas.

**Table 2:** Effect of sealed antennas on oviposition deterrent activities of Rhodojaponin-III against *B.dorsalis*.

Treatment	Number of eggs on the Rhodojaponin-III treated banana	Number of eggs on the untreated banana (water as control)	Oviposition deterrence rate (%)
Flies with wax sealed antennas	24.39±7.24	87.53±10.14	72.41±2.75a
Untreated flies	23.78±6.72	94.04±8.67	74.47±4.32a

Mean ± SD within columns followed by the same letter do not differ significantly using Tukey’s test, *P*< 0.05.

### Sequence analysis of BdorCSP2

**Figure 1:**
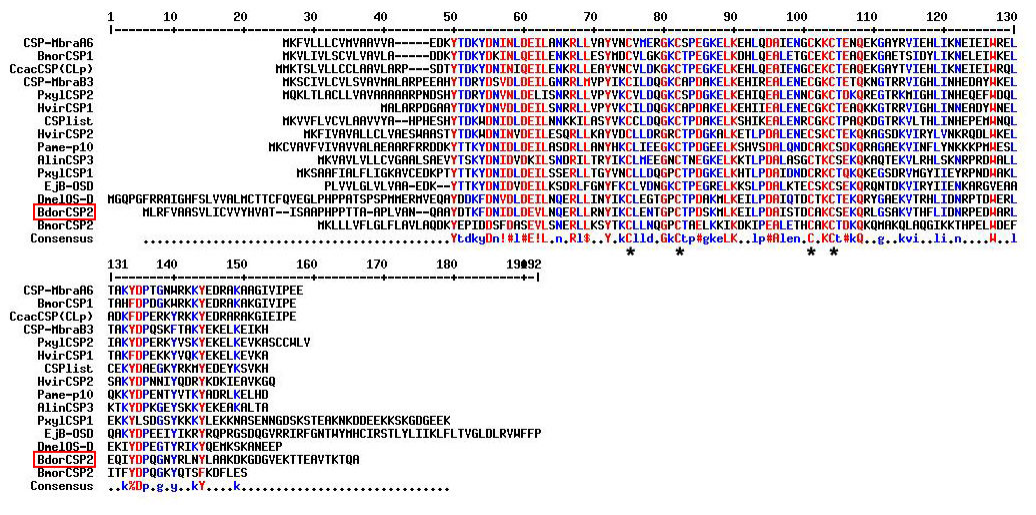
Alignment of peptide sequences of BdorCSP2 with those of the same class reported in other insect species. Full-length amino acid sequences are aligned by Clustal X. Red and blue colors showed the conserved residues in the alignment. Four conserved cysteines are marked by asterisks. The other insect species in the alignment are: CSP-MbraA6: *Mamestra brassicae* AAF71289.1; BmorCSP1: *Bombyx mori* AAV34688.1; CcacCSP(CLp): *Cactoblastis cactorum* AAC47827.1; CSP-MbraB3: *M. brassicae* AAF71289.1; PxylCSP2: *Plutella xylostella* ABM67687.1; HvirCSP1: *Heliothis virescens* AAM77041.1; CSPlist: *Spodoptera litura* AAY26143.1; HvirCSP2: *H. virescens* AAM77040.1; Pame-p10: *Periplaneta Americana* AAB84283.1; AlinCSP3: *Adelphocoris lineolatus* ACZ58021.1; PxylCSP1: *P. xylostella* ABM67686.1; EjB-OSD: *Drosophila melanogaster* AAA87058.1; DmelOS-D: *D. melanogaster* AAA21358.1; BmorCSP2: *B. mori* ABF51278.1.

**Figure 2:**
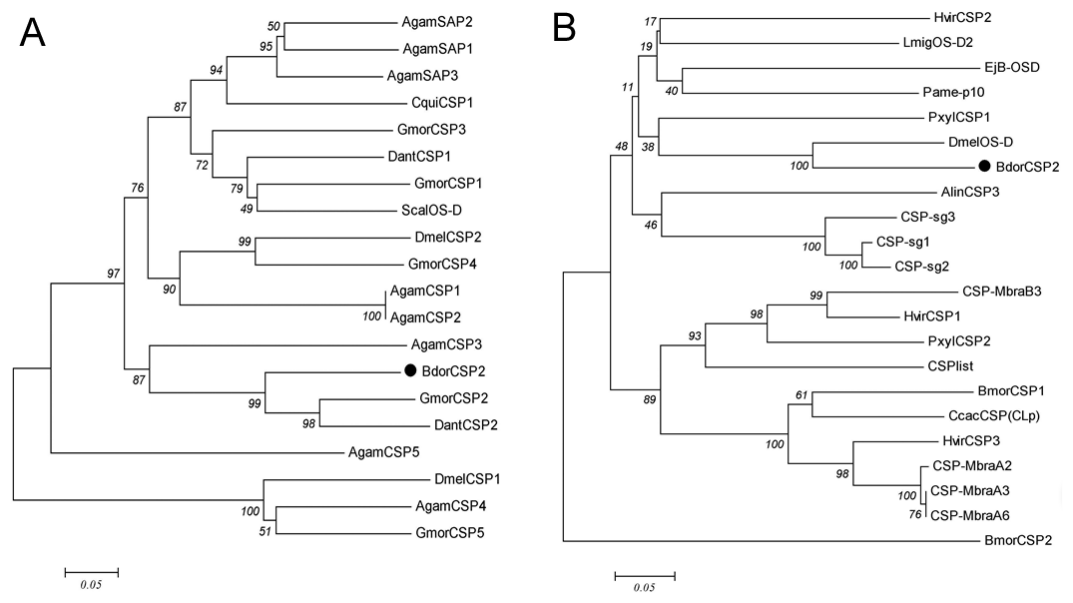
Phylogenetic trees of the deduced protein sequences of the CSPs in insects from the same order Diptera (A) and some contact sensilla (B). The tree was made by the neighbor-joining method with multiple alignments of amino acid sequences. Bootstrapping supports are indicated beside the branches at 1000 simulations. The sequences used in same order in Diptera (A), DmelCSP1: *D. melanogaster* CAG26928.1; DmelCSP2: *D. melanogaster* CAG26929.1; AgamCSP1: *A. gambiae* CAG26923.1; AgamCSP2: *A. gambiae* CAG26924.1; AgamCSP3: *A. gambiae* CAG26925.1; AgamCSP4: *A. gambiae* CAG26926.1; AgamCSP5: *A. gambiae* CAG26927.1; CquiCSP1: *Culex quinquefasciatus* XP_001844694.1; AgamSAP2: *A. gambiae* XP_317407.1; AgamSAP3: *A. gambiae* XP_317405.3; AgamSAP1: *A. gambiae* AAL84186.1; GmorCSP4: *Glossina morsitans* CBA11330.1; GmorCSP2: *G. morsitans* CBA11328.1; GmorCSP5: *G. morsitans* CBA11331.1; GmorCSP3: *G. morsitans* CBA11329.1; GmorCSP1: *G. morsitans* CAJ01495.1; ScalOS-D: *Stomoxys* *calcitrans* ACO83220.1; DantCSP1: *Delia* *antique* BAI82449.1; DantCSP2: *D. antique* BAI82450.1. The sequences used from the contact sensilla (B), CSP-MbraA2: *M. brassicae* AAF19648.1; CSP-MbraA3; *M. brassicae*AAF71289.1; CSP-MbraB3: *M. brassicae* AAF71289.1; HvirCSP1: *H. virescens* AAM77041.1; HvirCSP2: *H. virescens* AAM77040.1; HvirCSP3: *H. virescens* AAM77042.1; CSPlist: *S. litura* AAY26143.1; PxylCSP1: *P. xylostella* ABM67686.1; PxylCSP2: *P. xylostella* ABM67687.1; BmorCSP1: *B. mori* AAV34688.1; BmorCSP2: *B. mori* ABF51278.1; LmigOS-D2: *Locusta migratoria* CAB65178.1; AlinCSP2: *A. lineolatus* ACZ58021.1; EjB-OSD: *D. melanogaster* AAA87058.1; Pame-p10: *Periplaneta Americana* AAB84283.1; CcacCSP(CLp): *Cactoblastis cactorum* AAC47827.1; DmelOS-D: *D. melanogaster* AAA21358.1; CSP-sg1: *Schistocerca gregaria* AAC25399.1; CSP-sg2: *S. gregaria* AAC25400.1; CSP-sg3: *S. gregaria* AAC25401.1.

Based on the conserved sequences of CSPs from contacting chemosensory organs, the full length of BdorCSP2 was obtained (GenBank accession number: KC897022). The isolated cDNA encoding BdorCSP2 was 782 bp which contained a 471 bp open reading frame encoding a mature polypeptide with 135 amino acids and a signal peptide of the initial 22 amino acids. The predicted amino acid sequence of BdorCSP2 has the typical four-cysteine signature at N-terminus [46]. Multiple sequences alignment revealed that BdorCSP2 shared relatively high sequence identity with other members of the CSP family isolated from contact sensilla (45%-65%) (Figure 1). Two phylogenetic trees were constructed. For other species, the sequence analysis of CSPs (or SAPs) identified from *Drosophila melanogaster*, *Anopheles gambiae*, *Glossina morsitans*, *Stomoxys calcitrans*, *Delia antique* and *Culex quinquefasciatus*, as well as *B. dorsalis* showed that many CSPs within species are different (e.g. GmorCSP1, CSP2, CSP3, CSP4 and CSP5) (Figure 2A). This is consistent with the view that this family of genes arose by duplication events prior to the separation of the species and then diverged in each species. However, in terms of CSPs from the special tissues, some sequences were very similar even in different species (Figure 2B), which suggested that they may share common ancestral protein.

### Expression profiles of BdorCSP2

**Figure 3:**
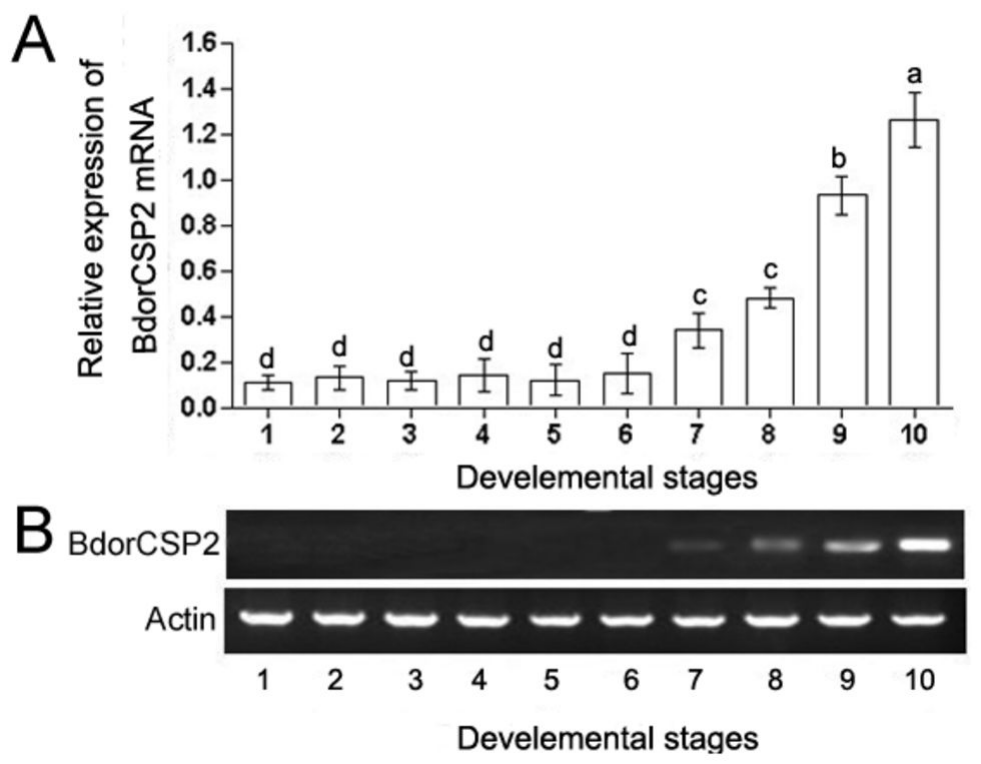
Expression of BdorCSP2 gene at different developmental stages was determined by qRT-PCR (A) and RT-PCR (B). The mRNA level was normalized relative to the β-Actin transcript. 1: Egg; 2: 1st instar larva; 3: 2st instar larva, 4; 3st instar larva; 5: 1 d-old pupa; 6: 4 d-old pupa; 7: 7 d-old pupa; 8: 10d-old pupa; 9: Newly-emerged male adult; 10:Newly-emerged female adult. Each point represented the mean value ± S.E.M of three independent experiments with three individuals in each replicate. Different letters indicated significant difference of the expression level of BdorCSP2 (*p* < 0.05, Duncan’s test).

**Figure 4:**
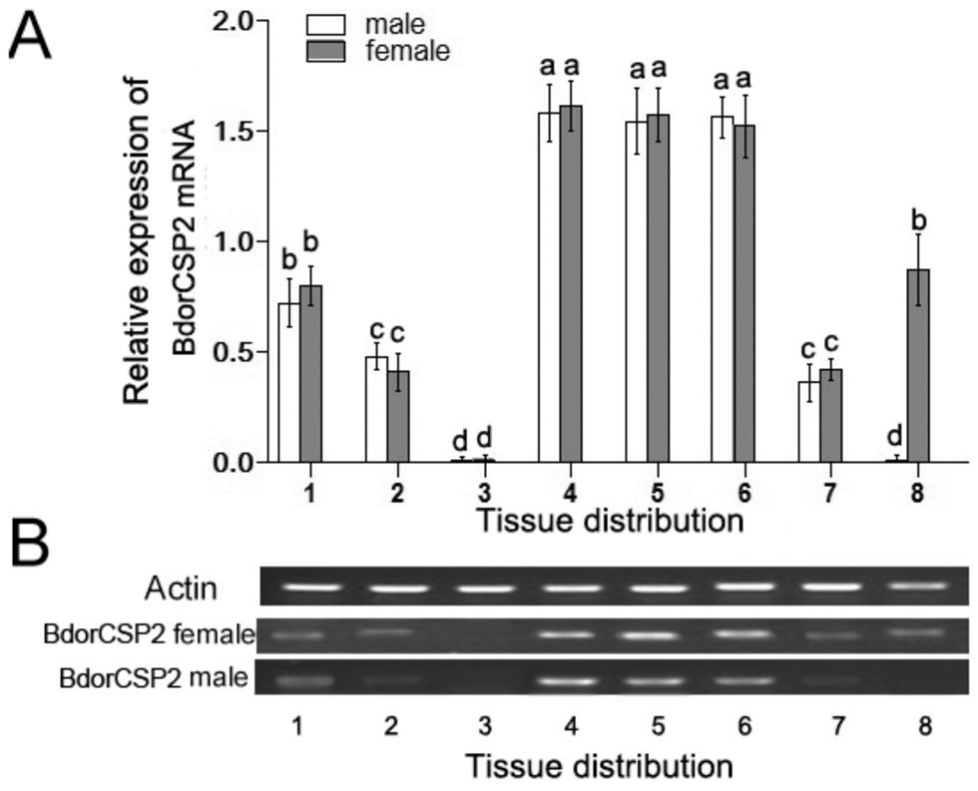
Expression of BdorCSP2 in various tissues was determined by qRT-PCR (A) and RT-PCR (B). The mRNA level was normalized relative to the β-Actin transcript. 1: Antenna; 2: Head (with antennae removed); 3: Thorax; 4: Fore legs; 5: Middle legs; 6: Hind legs; 7: Wings; 8: Genital segments. Each point represented the mean value ± S.E.M of three independent experiments with three individuals in each replicate. Different letters indicated significant difference of the expression level of BdorCSP2 (*p* < 0.05, Duncan’s test).

To examine developmental and tissue specific expression of BdorCSP2, its expression level was analyzed by qRT-PCR and RT-PCR. All samples were normalized to actin reference gene. The results of RT-PCR were consistent with the qRT-PCR. In the temporal expression pattern, BdorCSP2 was expressed significantly higher at the adult stage and late stages of pupae (Figure 3). The tissue distribution showed BdorCSP2 was mainly expressed in male and female legs (Figure 4). Additionally, other relatively high expression levels were observed in antennas, heads (with antennas removed), wings and genital segments (only in females). Collectively, the tissue distribution and temporal expression patterns revealed that the BdoCSP2 identified from legs of *B.dorsalis* appeared to be abundantly expressed in contacting chemosensory organs at adult stage.

### BdorCSP2 was upregulated by Rhodojaponin-III

**Figure 5:**
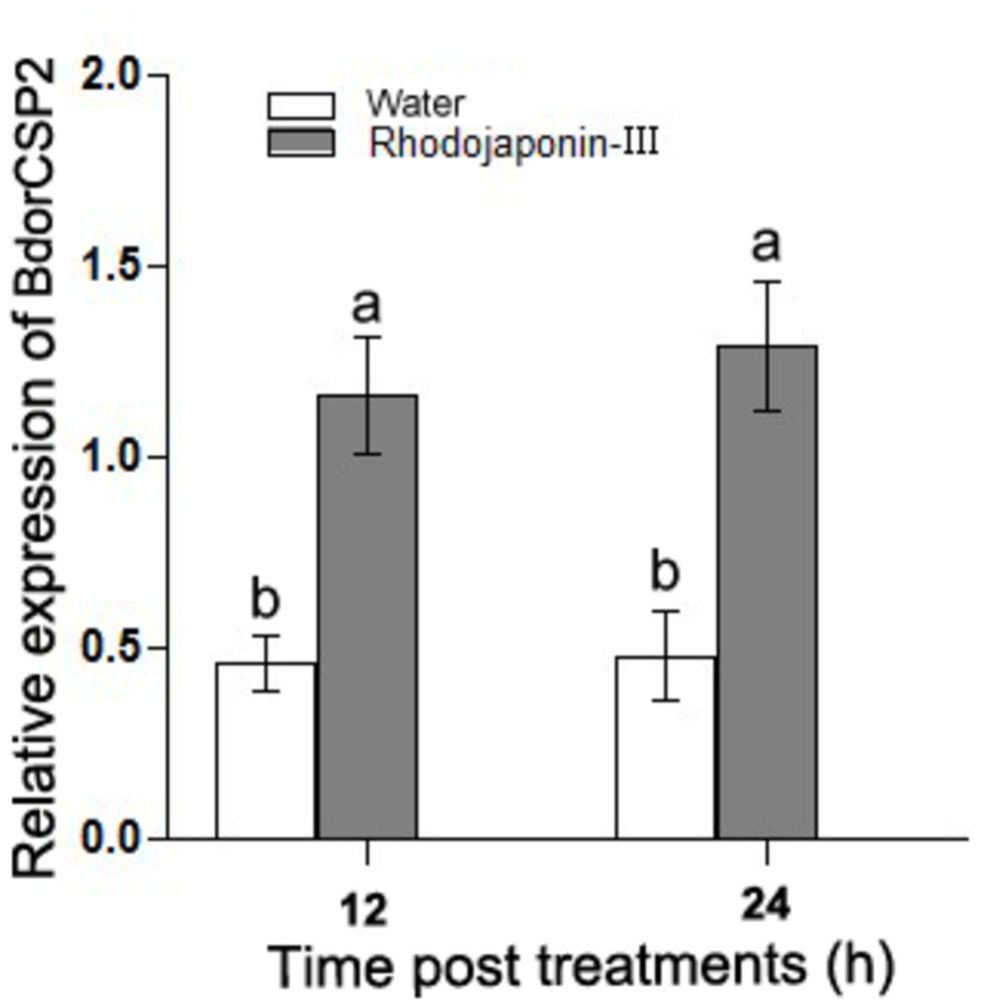
The effect of Rhodojaponin-III on BdorCSP2 expression was determined by qRT-PCR. Effect of 100 mg/L Rhodojaponin-III on the expression level of BdorCSP2 was detected by qRT-PCR after 12 h and 24 h treatment, respectively. Water treated was used as control. Different letters indicated significant difference in the expression level of BdorCSP2 (*p* < 0.05, Duncan’s test). Three biological replicates were performed.

To examine the effects of Rhodojaponin-III on BdorCSP2 expression, mature flies were placed into boxes and fed with bananas treated by Rhodojaponin-III. The results of qRT-PCR showed that the expression of BdorCSP2 in the flies was upregulated (2.7 folds compared to the control group) after treatment for 12 h (Figure 5). The results suggested that BdorCSP2 could be up-regulated by Rhodojaponin-III. 

### Fluorescence binding assays

**Figure 6:**
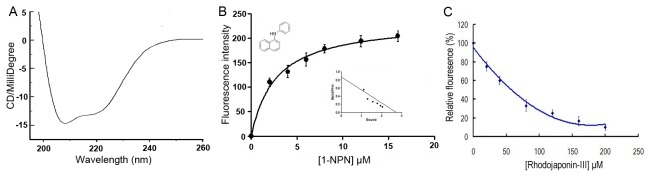
Competitive binding study of BdorCSP2 and Rhodojaponin-III. A, CD spectra of BdorCSP2. Spectra were recorded in 50 mM Na phosphate, pH 7.4, at room temperature between 190 and 260 nm. Datum are average value of three independent measurements. B, The binding curve of 1-NPN and relative Scatchard plot analysis (inset). To measure the affinity of 1-NPN to BdorCSP2, the fluorescence of 2 μM 1-NPN in 50 mM Tris-HCl was excited at 337 nm and emission spectra were recorded between 350 nm and 550 nm. And then, 2 μM of protein was added and titrated with aliquots of 1 mM 1-NPN to final concentrations of 2 to 16 μM. The experiment was replicated for at least three times, and the data were analyzed using Prism software and indicated the presence of a single binding site. The dissociation constant was 2.707 μM (±0.11 SEM). C, Competitive binding of the Rhodojaponin-III with BdorCSP2. Final solutions of 2 μM Bdor-CSP2 protein and 2 μM 1-NPN were titrated with Rhodojaponin-III in methanol to final concentrations of 0-200 μM. Datum arean average values of three independent measurements and error bars represented the standard deviations of the mean derived from the differences between the measurements.

As Figure 6A showed, this protein had one positive ellipticity at 192 nm and two negative ellipticities at 208 and 222 nm, which were typical of a fold with a majority of α-helical secondary structure. Since CD cannot provide information on the 3D structure, but only on the secondary structure content suggested the BdorCSP2 under study displayed a good overall fold. The BdorCSP2, which was purified and His-tag-removed, could be used for further investigation of its binding characteristics. This protein solution of 6.8 mg/mL was used to analyze the binding affinity of BdorCSP2 with Rhodojaponin-III. The fluorescence emission spectra showed maximally relative fluorescence intensity at 369 nm for BdorCSP2. 1-NPN was used as fluorescent probe to evaluate the binding ability in competitive binding experiment [47]. Figure 6B reported the BdorCSP2 could bind to 1-NPN with a dissociation constant of 2.707 ± 0.11 μM (Figure 6B). By titrating BdorCSP2 with increasing concentration of 1-NPN, a saturation and linear Scatchard plot were observed (Figure 6B), which indicated a single binding site. The IC_50_ value was 60.07 μM and calculated binding constant was 34.55 μM. The results showed that the good affinity between Bdor-CSP2 and Rhodojaponin-III was observed by using 1-NPN as the fluorescent reporter (Figure 6C). 

### Efficiency analysis after ingestion of dsBdorCSP2

After feeding synchronous emergence groups with 1 μg/μL dsBdorCSP2, dsGFP and DEPC water after 14 d, the survival rates were 83.21%, 87.85%, and 86.47%, respectively. To investigate the efficiency of RNAi after ingestion of dsRNA in *B. dorsalis*, BdorCSP2 mRNA levels were measured by qPCR at 2, 4 and 6 d after the ingestion, respectively. And the BdorCSP2 was investigated by Western blot at 6 d after ingestion.

**Figure 7:**
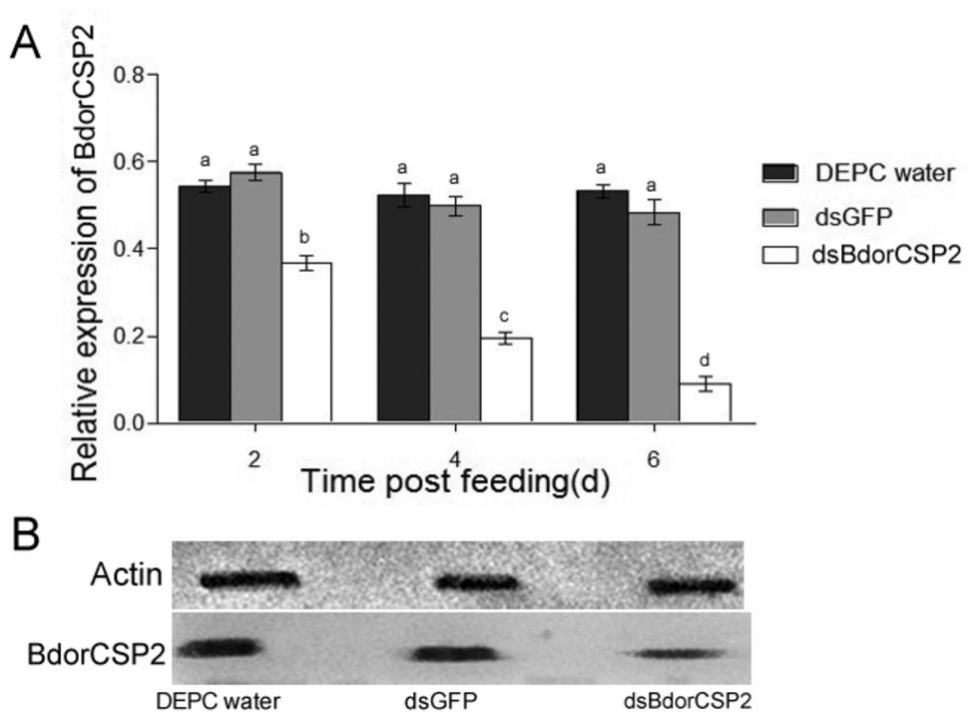
Efficiency analysis of RNAi after ingestion of dsRNA. Detection of the impact of RNAi on BdorCSP2 mRNA and protein levels after ingestion of dsRNA by RT-qPCR and Western blot, respectively. (A) The relative expression levels of BdorCSP2 mRNA after different treatments by ingestion dsRNA. Different letters indicated significant difference of the expression level of BdorCSP2 (*p* < 0.05, Duncan’s test). Three biological replicates were performed. (B) Western Blot analysis. Immunoblotted with serum (diluted 1: 2000) and visualized by ECL. Actin was used as an internal control. 1, DECP water; 2, dsGFP RNA; 3, dsBdorCSP2 RNA.

After 2-6 d ingestion of dsBdorCSP2, the expressions of BdorCSP2 decreased by 38.76-78.54% compared to the DEPC water and dsGFP treated groups. The band in Western blot was considerably more intense in the DEPC water and dsGFP treated groups than in the dsBdorCSP2-treated group, while the actin bands showed no change (Figure 7). These results confirmed that RNAi-mediated silencing of BdorCSP2 was highly effective.

### Silencing of BdorCSP2 resulted in disoriented physiological behaviors

**Figure 8:**
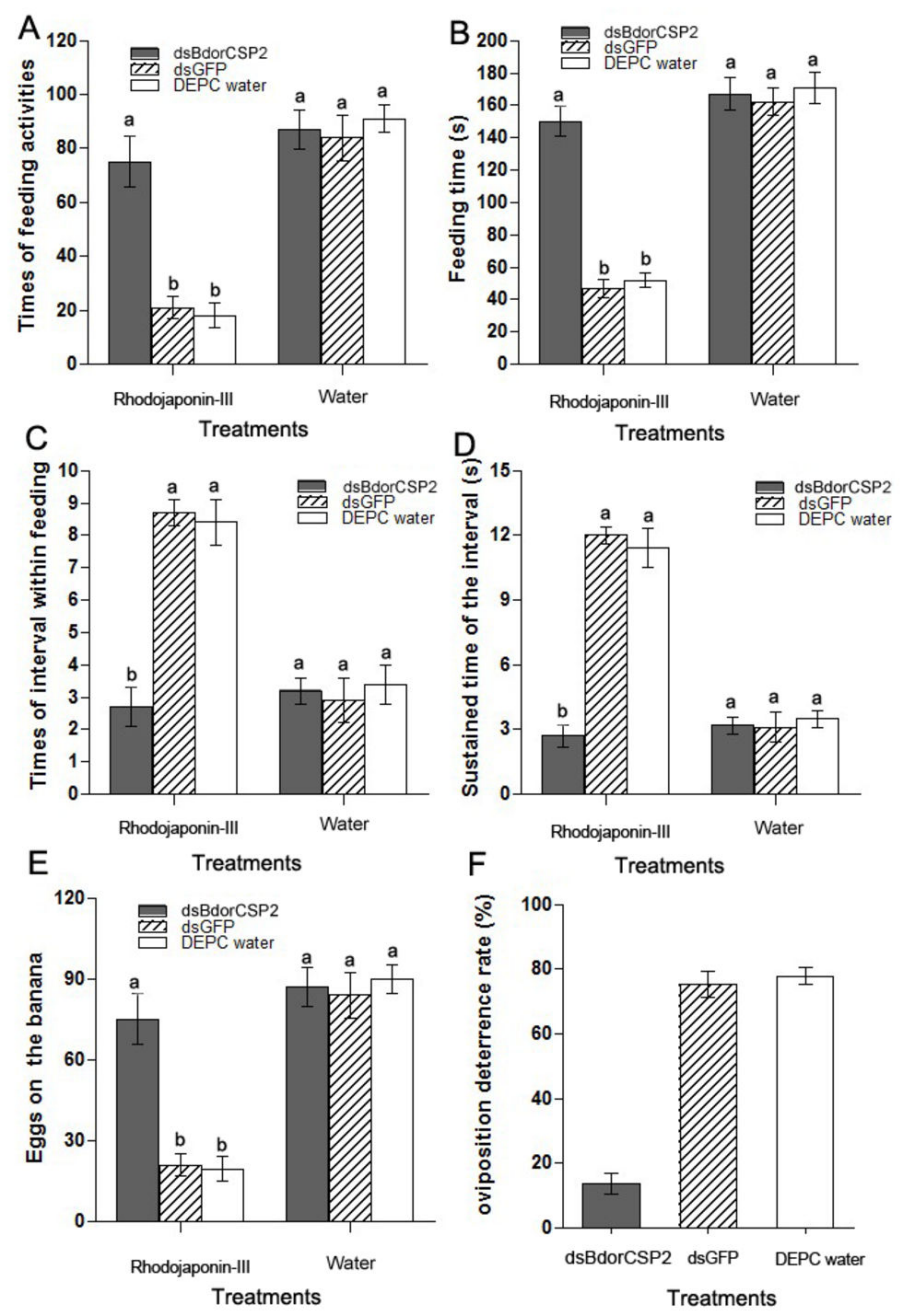
Roles of BdorCSP2 in antifeeding and oviposition deterring activities induced by Rhodojaponin-III. (A) The effect of Rhodojaponin-III on the frequency of feeding activity after silenced BdorCSP2 by RNAi. dsGFP and DEPC water were used as control. In the groups with bananas treated by Rhodojaponin-III, dsBdorCSP2 treated flies showed an increase in feeding frequency compared with the DEPC water and dsGFP treatment. (B) The effect of Rhodojaponin-III on the feeding time after silenced BdorCSP2 by RNAi. dsGFP and DEPC water were used as control. In the groups with bananas treated by Rhodojaponin-III, flies treated by dsBdorCSP2 showed longer average feeding time. (C) The effect of Rhodojaponin-III on the times of interval within feeding activity after silenced BdorCSP2 by RNAi. dsGFP and DEPC water were used as control. In the groups with bananas treated by Rhodojaponin-III, flies treated by dsBdorCSP2 showed a decrease in times of interval within feeding activity (D) The effect of Rhodojaponin-III on the sustained time of the interval feeding activity after silenced BdorCSP2 by RNAi. dsGFP and DEPC water were used as control. In the groups with bananas treated by Rhodojaponin-III, flies treated by dsBdorCSP2 showed less sustained time of the interval. (E) The effect of Rhodojaponin-III on the oviposition activity after silenced BdorCSP2 by RNAi. dsGFP and DEPC water were used as control. In the groups with bananas treated by Rhodojaponin-III, flies treated by dsBdorCSP2 showed an increase in oviposition. (F) The effect of Rhodojaponin-III on the oviposition deterrence rate after silenced BdorCSP2 by RNAi. dsGFP and DEPC water were used as control. In the dsBdorCSP2 group, the rate of oviposition deterring exerted by Rhodojaponin-III was significantly reduced compared with those treated by dsGFP and the DEPC water. The bananas treated by water were kept as control. All the data represent the mean values ± S.E.M of replicates. Different letters indicated significant differences of physiological behaviors between the treated by RNAi and control (those treated with dsGFP and the DEPC water) measured on the same banana groups, as determined using a *t*-test (*p*< 0.05).

In order to investigate the role of BdorCSP2 in recognition of Rhodojaponin*-*III, the bioassays were carried out. In the antifeedant test, flies treated by dsBdorCSP2 exhibited higher feeding frequency (4.8 fold, 5.2 fold) and longer feeding time (3.3 fold, 2.9 fold) compared with the DEPC water and dsGFP treatment (Figure 8A & Figure 8B). Besides, since the feeding behaviors of insects were not continuous, temporal interval time within feeding exists in every feeding activity. To obtain more precise evaluation, factors such as times of interval within feeding and sustained time of the interval could be taken as indicators to show effects of antifeedant behaviors. In our experiments, flies treated by dsBdorCSP2 showed fewer times of interval within feeding and less sustained time of the interval compared with the DEPC water and dsGFP treatment (Figure 8C & Figure 8D). Additionally, in the oviposition test, flies treated by dsBdorCSP2 resulted in 59.75% and 62.42% decrease in oviposition deterrence rate compared to those treated with dsGFP and the DEPC water, respectively (Figure 8E & 8F). The results suggested that BdorCSP2 played an important role in antifeeding and oviposition deterrence activities induced by Rhodojaponin-III against *B. dorsalis*.

## Discussion

In the repellent assay, the results showed the Rhodojaponin-III could still exert antifeeding and oviposition deterrence effects against the flies with antennas sealed. This result suggested the antennas did not really perform roles, or at least not the major target for a non-volatile repellent, like Rhodojaponin-III. During this experiment, we also observed that in order to locate the feeding and oviposition sites on bananas, the flies contacted the bananas with their legs or ovipositors tentatively. Collectively, these results showed that the flies might not perceive the active compound through the olfactory system on antennas, instead, the Rhodojaponin-III may exert its antifeedant and oviposition-deterring activities against flies by contacting chemosensation.

From legs of *B. dorsalis*, the full-length of BdorCSP2 cDNA was cloned by RT-PCR and RACE-PCR techniques. The general name of CSP was adopted for this class of proteins, followed by the initial of the species and a serial number. The result showed that BdorCSP2 shared relatively high sequence identity with other members of CSPs family (Figure 1) [46,48]. The phylogenetic analysis (Figure 2) indicated that different CSPs might perform similar role in insects. Thus, we cannot exclude the presence of other members in this class of proteins from *B. dorsalis*. In fact, studies have suggested a microdiversity of these proteins. The strong selective pressure on CSP orthologs indicated the structural conservation and their conserved functions in the insect chemoreception. An inherent binding preference determined by this conserved structure could help identify the ligand responses [49]. Therefore, as long time suspected and recently demonstrated by a number of relevant studies, CSPs were capable of reversibly binding with a great variety of organic molecules to cross an aqueous barrier [50]. 

The expression profiles of CSPs suggested they could be involved in insect olfaction, chemical perception and other novel functions. The BdorCSP2 was highly expressed in late pupae, which implied the function of BdorCSP2 in chemoreception and maintaining particular life activities [17]. High expression of BdorCSP2 in adult implied more olfactory receptor neurons (ORNs) in the adult and the essential roles of sense organs for adults to enable the flies to attract a spouse for courtship or to find the proper hosts for oviposition after mating (Figure 3) [44]. Tissue distribution showed BdorCSP2 was highly expressed in legs, which suggested this CSP might involve in feeding, oviposition activities and other contacting chemoreception processes via legs [12]. Our result was consistent with previous study that CSPs were expressed in some putative contact chemosensory sensilla in *Schistocerca gregaria*. They also reasoned that some specific CSPs could play roles in contacting chemoreception [18]. 

High degrees of binding specificities of CSPs and other ligands *in vitro* have been well established by fluorescent binding assay [32,43]. Our result showed the BdorCSP2 has good binding affinity with Rhodojaponin-III. In addition, the expression of Bdor-CSP2 was up-regulated when the flies were treated by Rhodojaponin-III. The good binding activity between BdorCSP2 and Rhodojaponin-III supported the hypothesis that this up-regulation of BdorCSP2 could be closely related to the recognition of Rhodojaponin-III by BdorCSP2 (Figure 6). Previous studies demonstrated that gene silencing could be successfully achieved by feeding dsRNA in *B. dorsalis* [36,44]. Experiments have demonstrated that silencing a single gene encoding protein of CSPs, OBPs or ORs, could abolish or modify electrophysiological and behavioral performance of the insects [51,52]. For our experiment, after silenced the expression of BdorCSP2, the *B. dorsalis* failed to distinguish the deterrent ability of Rhodojaponin-III, which led to disorientation in the feeding behavior and deterring activity of oviposition. The direct and reasonable explanation for this attributed to the significance of BdorCSP2 in recognizing Rhodojaponin-III. However, because there are many binding proteins in the chemoreceptive system, the detailed mechanism of signal transduction still requires further study.

The molecular mechanism of chemoreception in insects is complex, which comprised numerous classes of proteins and effectors to translate external stimulus in the environment to behavioral response in insects. Since chemical stimuli or plant compounds could drive specific insect behaviors such as mating, oviposition and feeding, isolating particular component which is responsible for the stimulus recognition could foster the development of novel insect control products [53]. In this work, the ligand-binding specificity of BdorCSP2 and Rhodojaponin-III was confirmed by the competitive binding experiment. In combination with RNAi, the study demonstrated that BdorCSP2 was essential for the recognition of Rhodojaponin-III, which could induce oviposition deterrence and antifeeding of *B. dorsalis*. Collectively, the essential characteristic and physiological significance of BdorCSP2 in recognizing Rhodojaponin-III and regulating Rhodojaponin-III-induced behaviors against *B. dorsali* were systematically investigated *in vitro* and *in vivo* in this paper. The molecular analysis and high affinity binding of BdorCSP2 and Rhodojaponin-III described here in combination with behavioral assays supported the functional roles of BdorCSP2 in the perception of nonvolatile secondary plant substance. This study provided molecular basis for the comprehensive understanding of antifeedant and oviposition deterring activities of Rhodojaponin-III against *B. dorsali.*
